# Effects of mutations conferring pyrazinamide resistance among multidrug-resistant tuberculosis isolates from China

**DOI:** 10.1128/spectrum.00641-26

**Published:** 2026-05-20

**Authors:** Xiang-long Bo, Ma-chao Li, Hao Hang, Shi-qiang Lin, Hai-can Liu, Cheng Qian, Gui-lian Li, Xiu-qin Zhao, Kang-Lin Wan, Li-li Zhao

**Affiliations:** 1 National Key Laboratory of Intelligent Tracking and Forecasting for Infectious Diseases, National Institute for Communicable Disease Control and Prevention, Chinese Center for Disease Control and Prevention 96698, Beijing, China; 2 College of Life Science, Fujian Agriculture and Forestry University 12449, Fuzhou, China; 3 Beijing Center for Disease Control and Prevention 661283 , Beijing, China; Maryland Department of Health 1496, Baltimore, Maryland, USA

**Keywords:** multidrug-resistant tuberculosis, pyrazinamide resistance, mutation, gene

## Abstract

To investigate the effects of mutations on pyrazinamide (PZA)-associated regions, including *pncA*, *rpsA*, *panD*, and *clpC1*, among multidrug-resistant tuberculosis (MDR-TB) isolates from China. A total of 139 MDR-TB isolates from China underwent PZA susceptibility testing and genotyping, and the sequences of the four PZA resistance-associated regions were analyzed. The association between lineage and PZA resistance was analyzed, as well as that between lineage and mutation. Among 139 MDR-TB isolates, 62 were resistant to PZA. Of these 62 PZA-resistant isolates, 60 harbored at least one nonsynonymous mutation in the *pncA*, *rpsA*, or *clpC1*. None of the isolates contained a mutation in *panD*. Most mutations in PZA-resistant isolates were concentrated in *pncA*, while mutations in other genes were rare and usually accompanied by mutations in *pncA*. The single mutation outside *pncA* observed in a PZA-resistant isolate was a novel *rpsA* mutation G(−131)A. The mutation Pro796Leu in *clpC1* was exclusively detected in lineage 4 strains and correlated with this lineage, but was not associated with PZA resistance. The DNA sequencing of *pncA* proved to be the most effective approach for detecting PZA resistance, with an accuracy of 92.81%. Notably, some PZA-susceptible isolates still carried mutations in *pncA*, *rpsA*, or *clpC1*. This study highlights the complexity of PZA resistance. These findings expand our understanding of the molecular features associated with PZA resistance in China.

**IMPORTANCE:**

In this study, we systematically investigated the mutations in pyrazinamide (PZA)-resistance-associated genes, including pncA, rpsA, panD, and clpC1, among the multidrug-resistant tuberculosis (MDR-TB) isolates from China, and their effects on the PZA resistance. This study is essential for comprehensively understanding the association between the phenotypic susceptibility to PZA and the mutation patterns in clinical MDR-TB isolates in China and is helpful for designing reliable and rapid molecular diagnostic assays for PZA resistance.

## INTRODUCTION

Tuberculosis (TB) remains a major public health threat. In 2023, there were an estimated 10.8 million new TB cases and 1.3 million deaths reported globally, with TB likely to regain its status as the world’s leading cause of death from a single infectious agent (1). The emergence of drug-resistant TB, particularly multidrug-resistant tuberculosis (MDR-TB), exacerbates this challenge: approximately 270,000 incident MDR-TB cases were reported in 2023 (2), and the treatment regimens for MDR-TB are notably more complex, costly, and time-consuming than those for drug-susceptible TB, while achieving lower efficacy.

Pyrazinamide (PZA), a key drug in first-line anti-tuberculosis treatment, is also commonly recommended for managing drug-resistant tuberculosis, including MDR-TB. This antibiotic plays a pivotal role in shortening the duration of anti-tuberculous treatment, as it exhibits potent activity against persistent tubercle bacilli under acidic pH conditions. Disturbingly, a relatively high proportion of MDR-TB isolates have demonstrated resistance to PZA. Hence, exploring the mechanisms underlying PZA resistance is crucial for developing reliable and rapid molecular diagnostic assays to guide the clinical use of this drug.

As a prodrug, PZA requires activation under acidic conditions by pyrazinamidase (PZase) (3–5). Encoded by *pncA*, PZase converts PZA to pyrazinoic acid (POA), which then disrupts mycobacterial activity (3, 4, 6). Numerous studies have shown that mutations in *pncA* contribute to PZA resistance in TB by impairing PZase function and reducing POA accumulation (6). However, some studies indicated that TB can develop resistance to PZA without mutations in *pncA* (7). These reports imply that additional mechanisms and/or genes may lead to PZA resistance in TB.

Ribosomal protein S1 (RpsA), encoded by the *rpsA* gene and involved in trans-translation, has been identified as a target of PZA. Mutations in *rpsA* can alter the binding of POA and have been detected in some PZA-resistant TB strains (8). More recently, mutations in *panD* and *clpC1*, encoding the aspartate decarboxylase PanD and the unfoldase ClpC1, have been observed to cause resistance to POA and PZA in *Mycobacterium tuberculosis* (9).

Notably, some mutations in PZA-resistance-associated genes, including *pncA*, *rpsA*, *panD*, and *clpC1*, have been detected in PZA-susceptible TB strains (10, 11). In addition, the absence of mutations in these four genes has also been observed in PZA-resistant TB strains (12). These contradictory results suggest that the mechanism of PZA resistance in TB is complex and requires more detailed investigation to clarify.

In this study, we systematically investigated the mutations in PZA-resistance-associated genes, including *pncA*, *rpsA*, *panD*, and *clpC1*, among the MDR-TB isolates from China, and their effects on PZA resistance. This study is essential for comprehensively understanding the association between the phenotypic susceptibility to PZA and the mutation patterns in clinical MDR-TB isolates in China and is helpful for designing reliable and rapid molecular diagnostic assays for PZA resistance.

## MATERIALS AND METHODS

### TB isolates

A total of 139 clinical MDR-TB isolates were collected from 139 patients with pulmonary TB across nine provinces in China, namely Fujian (17 isolates), Gansu (10 isolates), Guangxi (15 isolates), Guizhou (15 isolates), Hunan (17 isolates), Jilin (18 isolates), Inner Mongolia (19 isolates), Xinjiang (12 isolates), and Tibet (21 isolates). All strains were initially cultured on Lowenstein-Jensen medium and subsequently sub-cultured using the BACTEC MGIT 960 system (BD, Franklin Lakes, NJ, United States).

### Drug susceptibility testing for PZA

Drug susceptibility testing (DST) of all strains was conducted using the BACTEC MGIT 960 PZA kits at the recommended concentration of 100 μg/mL. H37Rv (ATCC 27294) was used for quality control in each batch of tests.

### Genome sequencing and bioinformatics analyses

Genomic DNA extraction, library construction, and sequencing data processing were performed as described previously (13). All nonsynonymous mutations within PZA resistance-associated genes (including *pncA*, *rpsA*, *panD*, and *clpC1*) and their upstream intergenic regions were analyzed. These sequencing data have been submitted to the NCBI Sequence Read Archive (SRA) under the accession number PRJNA1368812. Moreover, TB Profiler software (version 2.8.12) was utilized to predict the lineage of TB strains.

### PCR and DNA sequencing

All novel mutations identified in *pncA*, *rpsA*, *panD*, and *clpC1* were validated via PCR and subsequent DNA sequencing. The positions of the primer-amplified fragments are summarized in Table 1, including *pncA* (a 601 bp fragment containing the entire *pncA* coding region and 40 bp of the upstream region), *rpsA* (a 1,633 bp fragment encompassing the entire *rpsA* coding region, 162 bp of the upstream region, and 25 bp of the downstream region), *panD* (a 420 bp fragment containing the entire *panD* coding region), and *clpC1* (a 2,721 bp fragment containing the entire *clpC1* coding region and 174 bp of the upstream region). The sequencing data have also been uploaded to the NCBI SRA under the accession number PRJNA1368812.

**TABLE 1:** PCR primers for amplification and sequencing

Primer	Sequence (5′ to 3′)	Nucleotide position	TM^*a*^(℃)
*pncA*-F	GCGTCATGGACCCTATATC	2288604–2289269	58
*pncA*-R	AACAGTTCATCCCGGTTC		
*rpsA*-F	TCCACGACGAGCTGCTGTTC	1833219–1835022	58
*rpsA*-R	TGCGCAGCATTACGTGAACG		
*panD*-F	AATCGGAACTTTCGCCGG	4043690–4044309	58
*panD*-R	CACAACGGTGTGGGTGTT		
*clpC1*-F	GTTCTGTTCAACGGCGAC	4039161–4040855	56
*clpC1*-R	TGCAGCTCTTCTTCCATC		

^*a*^ TM, melting temperature.

### Statistical analysis

All data analyses were performed using SAS (version 9.3) software (SAS Institute, Cary, NC). Frequencies, percentages, and ranges were calculated as appropriate. Groups were compared using the χ^2^ test, with statistical significance defined as a *P* < 0.05.

### Resolution of discrepant results

When discrepancies arose between DST and genome sequencing results for some isolates, the isolates were retested using both DST and PCR sequencing. Results were finally classified as discrepant if the repeated testing yielded consistent findings.

## RESULTS

### DST results

Of the 139 MDR-TB isolates, 62 were determined to be PZA resistant, and 77 were PZA susceptible by DST. The prevalence of PZA resistance among MDR-TB in this study was 44.6% (62/139).

### Lineage analysis

Lineage analysis showed that the predominant strains among the 139 MDR-TB isolates belonged to lineage 2 (74.8%, 104/139), with the remainder belonging to lineage 4 (25.2%, 35/139). Among the 62 PZA-resistant strains, 43 (69.4%) belonged to lineage 2, and 19 (30.6%) belonged to lineage 4, whereas among the 77 PZA-susceptible strains, 61 (79.2%) belonged to lineage 2, and 16 (20.8%) belonged to lineage 4. No significant association was identified between lineage type and PZA resistance (*P* = 0.18).

### Mutations of PZA-resistance-associated genes

Sequencing analysis revealed that a total of 81 MDR-TB isolates, including 60 PZA-resistant ones and 21 PZA-susceptible ones, harbored at least one non-synonymous mutation in pncA, *rpsA*, and *clpC1* (Table 2). Among these, 65 isolates (80.2%, 65/81) carried a single gene mutation, while only 16 isolates (19.8%, 16/81) had multiple gene mutations. None of the isolates contained a mutation in *panD*.

**TABLE 2:** MDR-TB isolates harboring mutations within the *pncA*, *rpsA*, and *clpC1* genes

*pncA* mutations	Amino acid change	*rpsA* mutations	Amino acid change	*clpC1* mutations	Amino acid change	No. of isolates	DST
A→G at −11						1	R
T→C at 11	Leu4Ser					1	R
T→G at 11	Leu4Trp					2	R
T→C at 14	Ile5Thr					1	R
C→T at 28	Gln10^*b*^					1	R
A→C at 29	Gln10Pro			G→A at 2,387	Pro796Leu	1	R
G→A at 34	Asp12Asn					1	R
T→G- at 37^*a*^	Phe13Val			G→A at 2,387	Pro796Leu	1	R
C→A at 42	Cys14^*b*^			G→A at 2,387	Pro796Leu	1	R
T→C at 56	Leu19Pro					1	R
A→G at 139	Thr47Ala					2	R
C→T at 160	Pro54Ser			C→T at 172	Gly58Ser	1	R
C→G at 160^*a*^	Pro54Ala					1	R
A→G at −11, C→G at 160^*a*^	Pro54Ala					1	R
C→T at 161	Pro54Leu			G→A at 2,387	Pro796Leu	1	R
T→C at 175	Ser59Pro					1	R
C→T at 185	Pro62Leu					1	R
T→C at 202	Trp68Arg					1	R
C→A at 206^*a*^	Pro69Gln					1	R
T→C at 214	Cys72Arg					1	R
A→C at 226	Thr76Pro					3	R
T→G at 254	Leu85Arg					1	R
T→C at 254	Leu85Pro	G→A at 193	Ala65Thr	G→A at 2,387	Pro796Leu	1	R
G→C at 274	Ala92Pro					1	R
T→C at 280	Phe94Leu					1	R
A→C at 310	Ser104Arg					2	R
T→G at 316^*a*^	Phe106Val					1	R
T→C at 355	Trp119Arg					1	R
G→T at 357	Trp119Cys			G→A at 2,387	Pro796Leu	1	R
G→C at 362	Arg121Pro					1	R
A→G at 407	Asp136Gly	G→A at −131^*a*^				1	R
T→C at 416	Val139Ala					1	R
A→C at 422	Gln141Pro			C→A at 1,414^*a*^	Asp472Tyr	1	R
A→C at 422	Gln141Pro			G→A at 2,387	Pro796Leu	1	R
A→C at 422	Gln141Pro					2	R
G→A at 436	Ala146Thr					2	R
A→G at 460	Arg154Gly					1	R
T→G at 464	Val155Gly					1	R
A→C at 478	Thr160Pro					1	R
T→C at 524	Met175Thr					1	R
T→G at 524	Met175Arg					1	R
G→T at 538	Val180Phe					1	R
ATCGT insertion at 20 and 21^*a*^	Frame shift					1	R
C deletion at 59	Frame shift	G→A at −131^*a*^				1	R
G deletion at 68	Frame shift					1	R
G insertion at 82 and 83^*a*^	Frame shift					1	R
T deletion at 104^*a*^	Frame shift					1	R
CG insertion at 114 and 115^*a*^	Frame shift					1	R
AGGCG deletion at 272-276^*a*^	Frame shift			C→T at 603^*a*^	Met201Ile	1	R
C→GT- at 305	Frame shift					1	R
G insertion at 417 and 418	Frame shift					1	R
A→G at −11						1	S
T→C at 26^*a*^	Val9Pro			G→A at 2,387	Pro796Leu	1	S
G→C at 50^*a*^	Gly17Ala					1	S
A→G at 139	Thr47Ala					1	S
A→G at 386^*a*^	Asp129Glu					1	S
A→G at 478	Thr160Ala			G→A at 2,387	Pro796Leu	1	S
		G→A at −131^*a*^				1	R
		A→G at 163	Ile55Val			1	S
		T→G at 272^*a*^	Leu91Arg			1	S
		A→C at 368^*a*^	Asp123Ala			1	S
		C→G at 1,247^*a*^	Ala416Gly	G→A at 2,387	Pro796Leu	1	S
		C→T at 1,379^*a*^	Ala460Val	T→G at 482^*a*^	Glu161Ala	1	S
		G→C at 1,382^*a*^	Gly461Ala			1	S
				G→A at 2,387	Pro796Leu	1	R
				G→A at −80^*a*^		1	S
				G→A at 2,387	Pro796Leu	8	S

^*a*^ Novel mutation; DST, drug susceptibility testing.

^*b*^ Termination codon.

A total of 64 isolates, including 58 PZA-resistant ones and six PZA-susceptible ones, harbored mutations in the entire pncA gene (Table 2), which revealed 52 distinct mutated types. Only one (1.9%, 1/52) mutated type was located in the promoter region, while the remaining 51 (98.1%, 51/52) were scattered throughout the *pncA* coding region. The majority (*n* = 43) of mutations were point mutations that resulted in putative regulatory changes or amino acid substitutions, but insertions or deletions causing amino acid frame shifts (*n* = 9) were also detected. The most frequent point mutations were observed at codons 54 (*n* = 4) and 141 (*n* = 4), followed by mutations at codons 47, 76, 11, and −11, with three variants each. Overall, 13 novel mutations were detected in pncA. Moreover, there were six PZA-susceptible isolates carrying a mutated *pncA* gene (Table 2).

Mutations in *rpsA* (including the upstream region and the entire coding region) were identified in four PZA-resistant isolates and six PZA-susceptible isolates. Among the four PZA-resistant isolates, only one carried a single G(−131)A mutation in *rpsA*. The remaining three isolates, including two with the *rpsA* G(−131)A mutation and one with Ala65Thr mutation, were also concomitantly associated with *pncA* mutations. The six PZA-susceptible isolates harbored mutations in the *rpsA* coding region, specifically Ile55Val, Leu91Arg, Asp123Ala, Ala416Gly, Ala460Val, and Gly461Ala. None of these isolates were accompanied by the *pncA* mutation.

For *clpC1*, the Pro796Leu mutation was the most prevalent, occurring in 19 isolates (8 PZA resistant and 11 PZA susceptible). Statistical analysis showed no association between this mutation and PZA resistance (*P* = 0.94). Additionally, three PZA-resistant isolates and two PZA-susceptible isolates harbored other variations in *clpC1*, including G(−80)A, Gly58Ser, Glu161Ala, Met201Ile, and Asp472Tyr (Table 2). Notably, all three PZA-resistant isolates with *clpC1* mutations co-occurred with additional *pncA* mutations. In contrast, PZA-susceptible isolates either carried the single *clpC1* mutation G(−80)A or a *clpC1* mutation (Glu161Ala) combined with another *rpsA* mutation.

### Mutations and lineages

The distributions of mutations in different genes according to different lineages were summarized in Table 3. There was no association between lineages and mutations in *pncA* or *rpsA* (*P* > 0.05). However, for *clpC1*, a nonsynonymous Pro796Leu mutation was present in 19 isolates, all of which belonged to lineage 4. Statistical analysis also indicated that this mutation was associated with lineage 4 (*P* = 0.000). After removing the Pro796Leu mutation, other mutations in the *clpC1* gene showed no correlation with lineages (*P* > 0.05).

**TABLE 3:** The correlation between lineages and mutations^*a*^

Locus	No. of isolates				*P* value
	L2		L4		
	M	NM	M	NM	
*pncA*	44	60	20	15	0.128
*rpsA*	8	96	2	33	0.694
*clpC1 Pro796Leu*	0	104	19	16	0.000^*c*^
*clpC1^b^*	2	102	3	32	0.193

^*a*^ M, mutation; NM, no mutation.

^*b*^ Not contain clpC1Pro796Leu mutation.

^*c*^ *P* < 0.001.

### Prediction of PZA-resistant tuberculosis based on DNA sequencing

Table 4 summarizes the concordance between phenotypic and genotypic tests for detecting PZA resistance using DNA sequencing based on different PZA resistance-associated genes. In this study, screening of *pncA* yielded the best prediction performance (92.81% accuracy). The inclusion of other genes, such as *rpsA* or *clpC*1, did not improve the prediction accuracy (Table 4). Notably, 13 isolates exhibited a distinct discrepancy: phenotypic susceptibility tests confirmed them as PZA susceptible, yet they harbored at least one mutation in a PZA resistance-associated gene.

**TABLE 4:** Performance of sequencing on *pncA*, *rpsA*, and *clpC1* in relation to pyrazinamide susceptibility^*b*^

Gene	No. of isolates	MGIT 960 results		Sensitivity (%)	Specificity (%)	Accuracy (%)
		R	S			
*pncA*	M	58	6	93.55	92.21	92.81
	NM	4	71			
*rpsA*	M	4	6	6.45	92.20	53.96
	NM	58	71			
*clpC1^a^*	M	3	2	4.84	97.40	56.12
	NM	59	75			
*pncA + rpsA*	M	59	12	95.16	84.42	89.21
	NM	3	65			
*rpsA + clpC^a^*	M	7	7	11.29	90.91	55.40
	NM	55	70			
*pncA + rpsA + clpC1^a^*	M	59	13	95.16	83.12	88.49
	NM	3	64			

^*a*^ Not contain *clpC1Pro796Leu* mutation.

^*b*^ R, resistant; S, susceptible; M, mutation; NM, no mutation.

## DISCUSSION

Previous studies demonstrated that the prevalence of PZA resistance among MDR-TB ranged from 31% to 85% (14–17). Consistent with these findings, the proportion of PZA resistance in the present study was 44.6%. Such a high prevalence of PZA resistance in MDR-TB underscored the importance of rapid detection of PZA resistance for the control of MDR-TB.

In line with previous reports (18, 19), our data also showed that lineage 2 was the most predominant lineage in China. However, this lineage was not associated with PZA resistance. Furthermore, only lineage 2 and lineage 4 strains were identified in this study.

It has been reported that PZA resistance is correlated with mutations in PZA-associated genes, including *pncA*, *rpsA*, *panD*, and *clpC1*, with the most common mutations occurring in *pncA* (7). Consistently, we observed that among 62 PZA-resistant isolates, 58 (93.54%) harbored mutations in *pncA*. However, our results also showed fewer mutations in *rpsA* and *clpC1*, and no mutation in *panD*, among PZA-resistant isolates. This implied that mutations in *pncA* remain the most critical factor for PZA resistance.

Similar to the previous reports, all mutations of *pncA* were distributed throughout the gene and its promoter regions (19). Most mutations in *pncA* (63.5%, 33/52) were concentrated at amino acid residues 3–12, 46–62, 68–85, 94–103, and 132–142, which are areas near the pyrazinamide’s active sites or metal ion binding sites. Some studies have shown a widespread presence of mutations in *pncA* among PZA-susceptible isolates (20). Our research also revealed that six PZA-susceptible isolates harbored six different mutations in *pncA*: A(−11)G, Val9Pro, Gly17Ala, Thr47Ala, Asp129Glu, and Thr160Ala. Among them, mutations A(−11)G and Thr47Ala fell into group 1 (associated with resistance) for PZA according to the mutation catalog recommended by the World Health Organization (WHO) (21). One possible explanation for these inconsistent results is heteroresistance, a phenomenon in which only a small proportion of the bacterial population is drug resistant, at a level insufficient to reach the critical concentration required for phenotypic detection. This phenomenon is common in tuberculosis and is considered an early, yet potentially critical step in the development of PZA resistance. Moreover, several novel mutations were identified in the *pncA* gene, which broaden our understanding of the mutations related to PZA resistance.

Although numerous studies have indicated that mutations in the *rpsA* gene are closely associated with PZA resistance (22), this is the first time that a single mutation G(−131)A in the non-coding region of the *rpsA* alone has been identified in a PZA-resistant isolate. This mutation was also accompanied by *pncA* mutations in two other PZA-resistant isolates. The actual function of this mutation requires further exploration.

The Pro796Leu mutation in *clpC1* has been reported to occur in PZA-resistant isolates (7). This mutation can alter the structural conformation of the ClpC1 protein and thereby affect the stability of the ClpC1 hexamer, which is required for its chaperone activity (23). However, in our study, the Pro796Leu mutation was found in both PZA-resistant and PZA-susceptible isolates. Interestingly, this mutation was observed exclusively in lineage 4 strains, making it more likely to be a specific marker for a certain subtype of lineage 4 strains rather than a PZA-resistant marker.

Our study also revealed that almost all mutations in *rpsA* or *clpC1* occurring in PZA-resistant isolates co-occurred with *pncA* mutations. Single mutations in *rpsA* or *clpC1*, and even combined mutations of both genes, occurred predominantly in PZA-susceptible strains. This indicates that mutations in these two genes might have compensatory effects in PZA resistance. In addition, mutations in *panD* were absent, potentially due to the relatively limited sample size in this study.

Compared with the phenotypic results, the accuracy of detecting PZA resistance via DNA analysis of the single *pncA* gene in our study was 92.81%. Incorporating *rpsA*, *clpC1*, or *panD* into the molecular diagnosis either left the test accuracy unchanged or even reduced it. Thus, DNA analysis of *pncA* achieved the best accuracy for diagnosing PZA resistance. Notably, there were still three PZA-resistant isolates with no mutation detected in the analyzed regions. This indicates that these isolates may either carry mutations outside the analyzed area or develop resistance through other mechanisms, such as efflux pump regulation (24), regulatory mutations outside coding regions (25), and epistatic interactions between resistance-associated genes (26).

This study has several limitations. First, although 139 MDR-TB isolates were included, the numbers of PZA-resistant (*n* = 62) and mutation-harboring isolates (*n* = 72) were relatively small. Second, only four known PZA resistance-associated genes and their upstream regions were analyzed, leaving some resistance phenotypes unexplained. Further studies with a substantial panel of clinical isolates, especially PZA-resistant isolates, and more extensive genomic screening will therefore be required in the future.

In conclusion, mutations in *pncA* are the most crucial for PZA resistance, as almost all PZA-resistant isolates had at least one mutation in this region. DNA sequencing of this gene could achieve the best diagnostic efficiency for PZA resistance. However, mutations in *rpsA* and *clpC1* are likely to play compensatory roles in PZA resistance: these mutations either occur alongside *pncA* mutations in PZA-resistant isolates or appear alone in PZA-susceptible isolates. Some novel mutations were also observed in this study. These results are helpful for expanding our understanding of mutations associated with PZA resistance in China and for developing rapid molecular diagnostic methods.

## Data Availability

All sequencing data supporting this study have been deposited in the NCBI Sequence Read Archive (SRA) under the accession number PRJNA1368812.

## References

[1] Goletti D, Meintjes G, Andrade BB, Zumla A, Shan Lee S. 2025. Insights from the 2024 WHO global tuberculosis report - more comprehensive action, innovation, and investments required for achieving WHO end TB goals. Int J Infect Dis 150:107325. doi:10.1016/j.ijid.2024.107325 39631498

[2] Liu B, Su P, Hu P, Yan M, Li W, Yi S, Chen Z, Zhang X, Guo J, Wan X, Wang J, Gong D, Bai H, Wan K, Liu H, Li G, Tan Y. 2024. Prevalence, transmission and genetic diversity of pyrazinamide resistance among multidrug-resistant *Mycobacterium tuberculosis* isolates in Hunan, China. Infect Drug Resist 17:403–416. doi:10.2147/IDR.S436161 38328339 PMC10849141

[3] Stehr M, Elamin AA, Singh M. 2015. Pyrazinamide: the importance of uncovering the mechanisms of action in *Mycobacteria*. Expert Rev Anti Infect Ther 13:593–603. doi:10.1586/14787210.2015.1021784 25746054

[4] Tamilzhalagan S, Justin ES, Selvaraj A, Venkateswaran K, Sivakumar AK, Chittibabu S, McLaughlin HP, Moonan PK, Smith JP, Suba S, Sathya Narayanan MK, Ho CS, Kumar N, Tripathy SP, Shanmugam SK, Hall-Eidson PJ, Ranganathan UD. 2024. Phenotypic and genotypic characterization of *Mycobacterium tuberculosis* pyrazinamide resistance-India, 2018-2020. Front Microbiol 15:1515627. doi:10.3389/fmicb.2024.1515627 39845030 PMC11750862

[5] Xia Q, Zhao L-L, Li F, Fan Y-M, Chen Y-Y, Wu B-B, Liu Z-W, Pan A-Z, Zhu M. 2015. Phenotypic and genotypic characterization of pyrazinamide resistance among multidrug-resistant *Mycobacterium tuberculosis* isolates in Zhejiang, China. Antimicrob Agents Chemother 59:1690–1695. doi:10.1128/AAC.04541-14 25583712 PMC4325781

[6] Li K, Yang Z, Gu J, Luo M, Deng J, Chen Y. 2021. Characterization of pnca mutations and prediction of pza resistance in *Mycobacterium tuberculosis* clinical isolates from Chongqing, China. Front Microbiol 11:587948. doi:10.3389/fmicb.2020.594171 PMC7832174 33505367

[7] Hameed HMA, Fang C, Liu Z, Gao Y, Wang S, Chen X, Zhong N, Aung HL, Hu J, Zhang T. 2025. The Identification of novel mutations in ATP-dependent protease ClpC1 assists in the molecular diagnosis of obscured pyrazinamide-resistant tuberculosis clinical isolates. Microorganisms 13:1401. doi:10.3390/microorganisms13061401 40572289 PMC12196252

[8] Shi W, Zhang X, Jiang X, Yuan H, Lee JS, Barry CE 3rd, Wang H, Zhang W, Zhang Y. 2011. Pyrazinamide inhibits trans-translation in *Mycobacterium tuberculosis*. Science 333:1630–1632. doi:10.1126/science.1208813 21835980 PMC3502614

[9] Gopal P, Sarathy JP, Yee M, Ragunathan P, Shin J, Bhushan S, Zhu J, Akopian T, Kandror O, Lim TK, Gengenbacher M, Lin Q, Rubin EJ, Grüber G, Dick T. 2020. Pyrazinamide triggers degradation of its target aspartate decarboxylase. Nat Commun 11:1661. doi:10.1038/s41467-020-15516-1 32245967 PMC7125159

[10] Wang N, Meng F, Deng L, Wu L, Yang Y, Li H, Chen Y, Wei Z, Xie B, Gong L, Niu Q, Lei J, Gao J, Huang B, Wang Q, Lai X, Liu Z, Hu J. 2025. The epidemiology and gene mutation characteristics of pyrazinamide-resistant *Mycobacterium tuberculosis* clinical isolates in Southern China. Emerg Microbes Infect 14:2447607. doi:10.1080/22221751.2024.2447607 39745172 PMC11721771

[11] Liu BB, Hu PL, Chen ZH, Yi SL, Zhang XP, Tan YH. 2022. Prevalence and transmission of pyrazinamide-resistant *Mycobacterium tuberculosis* in Hunan Province, China. Chinese journal of tuberculosis and respiratory diseases. 45:677–685. doi:10.3760/cma.j.cn112147-20211219-00904 35768376

[12] Shi J, Su R, Zheng D, Zhu Y, Ma X, Wang S, Li H, Sun D. 2020. Pyrazinamide resistance and mutation patterns among multidrug-resistant *Mycobacterium tuberculosis* from Henan Province. Infect Drug Resist 13:2929–2941. doi:10.2147/IDR.S260161 32903869 PMC7445508

[13] Li M-C, Wang W, Xiao T-Y, Liu H-C, Lin S-Q, Hang H, Bo X-L, Nan X-T, Qian C, Fan X-T, Zhao X-Q, Li G-L, Wan K-L, Zhao L-L. 2025. The implications of mutations in multiple genes associated with ethambutol resistance among multidrug-resistant tuberculosis isolates from China. BMC Microbiol 25:107. doi:10.1186/s12866-025-03821-y 40025456 PMC11871839

[14] Che Y, Bo D, Lin X, Chen T, He T, Lin Y. 2021. Phenotypic and molecular characterization of pyrazinamide resistance among multidrug-resistant *Mycobacterium tuberculosis* isolates in Ningbo, China. BMC Infect Dis 21:605. doi:10.1186/s12879-021-06306-1 34171989 PMC8228925

[15] Wang G, Jiang G, Jing W, Zong Z, Yu X, Chen S, Li W, Huang H. 2021. Prevalence and molecular characterizations of seven additional drug resistance among multidrug-resistant tuberculosis in China: a subsequent study of a national survey. J Infect 82:371–377. doi:10.1016/j.jinf.2021.02.004 33556430

[16] Park S, Jo KW, Shim TS. 2020. Treatment outcomes in multidrug-resistant tuberculosis according to pyrazinamide susceptibility. Int J Tuberc Lung Dis 24:233–239. doi:10.5588/ijtld.19.0314 32127109

[17] Wang Z, Tang Z, Heidari H, Molaeipour L, Ghanavati R, Kazemian H, Koohsar F, Kouhsari E. 2023. Global status of phenotypic pyrazinamide resistance in *Mycobacterium tuberculosis* clinical isolates: an updated systematic review and meta-analysis. J Chemother 35:583–595. doi:10.1080/1120009X.2023.2214473 37211822

[18] Liu D, Badeerhan G, Emam M, Jiang M, Hong G, Xie M, Liu Y, Ma L, Xu L, Wang X, Wei Q. 2025. Whole genome sequencing for tuberculosis disease species identification, lineage determination, and drug resistance detection in Kashgar prefecture, China. BMC Infect Dis 25:1239. doi:10.1186/s12879-025-11221-w 41057789 PMC12502365

[19] Gao W, Wang W, Li J, Gao Y, Zhang S, Lei H, He L, Li T, He J. 2024. Drug-resistance characteristics, genetic diversity, and transmission dynamics of multidrug-resistant or rifampicin-resistant *Mycobacterium tuberculosis* from 2019 to 2021 in Sichuan, China. Antimicrob Resist Infect Control 13:125. doi:10.1186/s13756-024-01482-6 39396971 PMC11472436

[20] Bhuju S, Fonseca L de S, Marsico AG, de Oliveira Vieira GB, Sobral LF, Stehr M, Singh M, Saad MHF. 2013. *Mycobacterium tuberculosis* isolates from Rio de Janeiro reveal unusually low correlation between pyrazinamide resistance and mutations in the pnca gene. Infect Genet Evol 19:1–6. doi:10.1016/j.meegid.2013.06.008 23770140

[21] World Health Organization. 2023. Catalogue of mutations in Mycobacterium tuberculosis complex and their association with drug resistance. 2nd ed. World Health Organization, Geneva,Switzerland.

[22] Shi W, Cui P, Niu H, Zhang S, Tønjum T, Zhu B, Zhang Y. 2019. Introducing rpsa point mutations Δ438A and D123A into the chromosome of *Mycobacterium tuberculosis* confirms their role in causing resistance to pyrazinamide. Antimicrob Agents Chemother 63:e02681-18. doi:10.1128/AAC.02681-18 30858213 PMC6535565

[23] Ogbonna EC, Anderson HR, Beardslee PC, Bheemreddy P, Schmitz KR. 2023. Interactome analysis identifies MSMEI_3879 as a substrate of *Mycolicibacterium smegmatis* ClpC1. Microbiol Spectr 11:e04548-22. doi:10.1128/spectrum.04548-22 37341639 PMC10433963

[24] Zhang Y, Zhang J, Cui P, Zhang Y, Zhang W. 2017. Identification of novel efflux proteins Rv0191, Rv3756c, Rv3008, and Rv1667c involved in pyrazinamide resistance in *Mycobacterium tuberculosis*. Antimicrob Agents Chemother 61:e00940-17. doi:10.1128/AAC.00940-17 28584158 PMC5527661

[25] Farhat MR, Freschi L, Calderon R, Ioerger T, Snyder M, Meehan CJ, de Jong B, Rigouts L, Sloutsky A, Kaur D, Sunyaev S, van Soolingen D, Shendure J, Sacchettini J, Murray M. 2019. Gwas for quantitative resistance phenotypes in *Mycobacterium tuberculosis* reveals resistance genes and regulatory regions. Nat Commun 10:2128. doi:10.1038/s41467-019-10110-6 31086182 PMC6513847

[26] Rehman A, Jeukens J, Levesque RC, Lamont IL. 2021. Gene-gene interactions dictate ciprofloxacin resistance in *Pseudomonas aeruginosa* and facilitate prediction of resistance phenotype from genome sequence data. Antimicrob Agents Chemother 65:e0269620. doi:10.1128/AAC.02696-20 33875431 PMC8218647

